# On the validity of the centrality hypothesis in cross-sectional between-subject networks of psychopathology

**DOI:** 10.1186/s12916-020-01740-5

**Published:** 2020-10-12

**Authors:** Tobias R. Spiller, Ofir Levi, Yuval Neria, Benjamin Suarez-Jimenez, Yair Bar-Haim, Amit Lazarov

**Affiliations:** 1 Department of Consultation-Liaison Psychiatry and Psychosomatic Medicine, University Hospital Zurich, Zurich, Switzerland; 2 Division of Mental Health, Medical Corps, Israel Defense Forces, Tel Aviv, Israel; 3 Social Work Department, Ruppin Academic Center, Emek Hefer, Israel; 4 Bob Shapell School of Social Work, Tel Aviv University, Tel Aviv, Israel; 5 Departments of Psychiatry, Columbia University Irving Medical Center, New York, NY USA; 6 New York State Psychiatric Institute, New York, NY USA; 7 School of Psychological Sciences, Tel Aviv University, Tel Aviv, Israel; 8 Sagol School of Neuroscience, Tel Aviv University, Tel Aviv, Israel

**Keywords:** Posttraumatic stress disorder, Network analysis, Network approach, Centrality measures, Treatment

## Abstract

**Background:**

In the network approach to psychopathology, psychiatric disorders are considered networks of causally active symptoms (nodes), with node centrality hypothesized to reflect symptoms’ causal influence within a network. Accordingly, centrality measures have been used in numerous network-based cross-sectional studies to identify specific treatment targets, based on the assumption that deactivating highly central nodes would proliferate to other nodes in the network, thereby collapsing the network structure and alleviating the overall psychopathology (i.e., the centrality hypothesis).

**Methods:**

Here, we summarize three types of evidence pertaining to the centrality hypothesis in psychopathology. First, we discuss the validity of the theoretical assumptions underlying the centrality hypothesis in psychopathology. We then summarize the methodological aspects of extant studies using centrality measures as predictors of symptom change following treatment, while delineating their main findings and several of their limitations. Finally, using a specific dataset of 710 treatment-seeking patients with posttraumatic stress disorder (PTSD) as an example, we empirically examine node centrality as a predictor of therapeutic change, replicating the approach taken by previous studies, while addressing some of their limitations. Specifically, we investigated whether three pre-treatment centrality indices (strength, predictability, and expected influence) were significantly correlated with the strength of the association between a symptom’s change and the change in the severity of all other symptoms in the network from pre- to post-treatment (Δnode-Δnetwork association). Using similar analyses, we also examine the predictive validity of two simple non-causal node properties (mean symptom severity and infrequency of symptom endorsement)*.*

**Results:**

Of the three centrality measures, only expected influence successfully predicted how strongly changes in nodes/symptoms were associated with change in the remainder of the nodes/symptoms. Importantly, when excluding the amnesia node, a well-documented outlier in the phenomenology of PTSD, none of the tested centrality measures predicted symptom change. Conversely, both mean symptom severity and infrequency of symptom endorsement, two standard non-network-derived indices, were found to be more predictive than expected influence and remained significantly predictive also after excluding amnesia from the network analyses.

**Conclusions:**

The centrality hypothesis in its current form is ill-defined, showing no consistent supporting evidence in the context of cross-sectional, between-subject networks.

## Background

The “network approach to psychopathology,” a collective term for theoretical, methodological, and empirical work conceptualizing psychiatric disorders as networks of causally interacting symptoms (i.e., nodes), reflective of complex systems, has become increasingly prominent over the last decade [1]. Specifically, according to this approach, psychopathology is not the result of an underlying latent variable responsible for causing the observant symptoms, but rather emerges from the dynamic and causal interaction among symptoms [2–7]. Thus, a presumably causal network of symptoms (“nodes”), and the connections between them (“edges”), establishes a specific disorder [8]. While key theoretical concepts and hypotheses underlying this approach have been outlined by several different contributors [2, 5, 6, 9–15], they all share the fundamental assumption that applying concepts and methods developed in “network science” will successfully lead to novel insights into the nature of psychopathology, yielding relevant and important clinical implications (e.g., [13]).

In this regard, high hopes were especially put in the concept of *node centrality* [11], an indicator of the importance of different nodes within a specific network [16]. Put differently, nodes’ centrality reflects their influence over other nodes in the network, or how relevant they are to the entire network structure, such that nodes with high centrality are considered to have above average influence on the rest of the network [2]. In empirical data, node centrality can be determined using several centrality metrics [6], including, among others, node strength, predictability, and expected influence (for more details see [17, 18]), with higher values reflecting greater node centrality/influence. More formally, *strength* is defined as to the sum of the absolute value of all edge weights of a node [17]. *Expected influence* is similar to strength, but takes the directionality (i.e., if an edge weight is negative or positive) into account by removing the usage of absolute values of edge weights when computing a node’s strength in favor of actual values [18]. *Predictability* is equal to the upper bound of the shared variance of a given node (measured in *R*^2^) with all its neighboring nodes, assuming that all connections are directed towards that given node [19]. Thus, strength and expected influence are both relative measures of node centrality, whereas predictability is considered a more “objective” centrality measure, as it can be compared across different networks. From a clinical standpoint, it has been argued that if central nodes within a psychopathology network represent highly causal influential symptoms, then the treatments specifically targeting these central nodes/symptoms should be more efficacious than other treatments that do not. Specifically, targeting highly central nodes to reduce their severity should propagate to other nodes in the network causally affected by them, thereby eventually collapsing the entire network structure and alleviating the overall psychopathology [2]. For example, if sleep quality is a central node causally affecting concentration and irritability, then enhancing sleep quality would also increase concentration abilities and reduce irritability. Indeed, the results of numerous empirical network studies in psychopathology have been interpreted in light of this stipulation (e.g., [20–23]), with some grounding the rational for conducting their studies, at least partially, on this claim (e.g., [24–27]).^1^ Hence, elucidating the validity of this hypothesis is of crucial importance for the network approach to psychopathology in general, and, more specifically, for its clinical significance and implications.

Here we summarize key theoretical, methodological, and empirical evidence pertaining to the centrality hypothesis. We focus on networks derived from cross-sectional between-subject data as most network research in psychopathology have used this kind of data [1, 30, 31], including prior empirical investigations specifically exploring the predictive validity of central nodes as treatment targets [18, 32–34]. We first introduce and discuss several theoretical limitations of the centrality hypothesis. We then summarize existing empirical evidence pertaining to the centrality hypothesis and discuss key methodological issues of extant research. Next, using a specific dataset as an example, we empirically test the centrality hypothesis by replicating the methods used by prior studies, while addressing some of their limitations. Specifically, we examine a sample of 710 treatment-seeking posttraumatic stress disorder (PTSD) veteran adult patients who completed a PTSD assessment, including both clinician-assessed and self-reported measures, before and after PTSD-specific treatments. Finally, we discuss the implications of our empirical results in light of the presented theoretical and methodological arguments for both researchers and clinicians working under the “network approach to psychopathology.”

### Theoretical aspects

The validity of any hypothesis is always built upon the validity of its underlying assumptions. Thus, here we will outline and examine the validity of some explicit and implicit assumptions underlying the centrality hypothesis in general, and, more specifically, in the context of networks based on cross-sectional between-subject data. In doing so, we assume that the critical hypothesis of the network approach, namely, that “symptoms may cohere as syndromes because of causal relations among the symptoms themselves” [1] is true and that this can indeed be modeled by network analytic methods.

A first fundamental underlying assumption of the centrality hypothesis is that centrality metrics reliably model the causal importance of individual nodes. This assumption, however, has been questioned on different grounds. First, commonly used centrality metrics stem from the field of social networks and it remains unclear whether centrality measures can be indeed effectively applied to complex networks describing psychopathology, as they are based on assumptions that seem implausible in relation to psychopathology [35]. For example, the nodes of a network are assumed to be fully interchangeable (i.e., that they are conceptually equivalent), which seems implausible when considering the clinical meaning of psychopathological symptoms. For instance, although suicidality and insomnia are both symptoms of a major depressive episode, their clinical meaning and implications differ significantly when estimating depression severity, prognosis, and treatment options. No clinician will consider the two substitutable. Thus, the assumption that nodes are fully interchangeable is clearly violated. Moreover, the conceptual validity of the developed centrality metrics has been doubted even in social network science (for more details see [34]). Second, a network, and thus its centrality measures, can only reflect true causal relations if all variables with a relevant causal effect are indeed included in the model [36], without omitting any important causal variables [8]. Currently, however, it seems highly implausible that all necessary causal effects of an examined psychopathology are even known, let alone included in the corresponding networks.

To assume the validity of the centrality hypothesis, a second fundamental assumption must be made, namely, that the abovementioned first assumption (i.e., that centrality metrics reliably model the causal importance of individual nodes) holds in any specific empirical context under which it is being used or examined. However, here, too, several discrepancies and inconsistencies arise. First, the assumption that symptoms causally interact with each other implies that they do so *within the individual* and *over time*, necessitating empirical methods which can recover these effects with the adequate precision. Considering the *within individual* requirement, the sufficient and necessary assumptions under which individual effects can be recovered from between-subject data settings, known as group-to-individual generalizability, are highly debated [37–40]. While some claim that generalizability is only possible if group effects are homogeneous across individuals (i.e., that they are ergodic—a process in which every sample is equally representative of the whole [37]), others consider ergodicity as a sufficient condition, questioning it as a necessary one [39] (For more details on this important debate, see [40–42]).^2^ Considering the *overtime* aspect of the causality assumption, research has shown that networks based on longitudinal data differ from networks based on the same “cross-sectionalized” data (e.g., by averaging the data, [43]; that some effects [e.g., temporal ones] can only be assessed in longitudinal data, [44]; and that centrality derived from a network based on longitudinal data does not correlate with centrality derived from a cross-sectional network based on the same averaged, longitudinal data, [45]). Second, while networks based on cross-sectional data and/or group-level analysis are most common [30, 46], some have used ideographically collected data to estimate centrality measures*.* However, a recent simulation study demonstrated that current network analytic methods are only partially successful in recovering the properties and dynamics of bi-stable systems (indicating a healthy and “sick” state) in a common ideographic research setting [47]. Third, results have shown that Gaussian graphical models, the most often used models to estimate networks based on cross-sectional between-subject data, to be incapable of differentiating several possible underlying causal models (i.e., directed acyclic graphs [48]), with centrality found to potentially reflect common endpoints (i.e., causal results) rather than causally important symptoms [1]. Finally, the methodological choices made during the process of estimating a data-driven network have a substantial influence on the resulting network structure and, hence, on the emerging centrality measures [49, 50].^3^ Moreover, even when following the same procedure outlined and implemented in an R package, instability of some centrality indices across studies still emerges [30].

In sum, theoretically-wise, it seems that centrality metrics are limited in their ability to reveal causally influential nodes [52]. In addition, standardized processing pipelines are highly needed to enable comparability of empirical results across studies. Taken together, the entirety of the theoretical assumptions and concerns challenges the validity of centrality measures in identifying symptoms constituting optimal treatment targets, especially in cross-sectional between-person networks, which have nevertheless dominated the network empirical research over the last several years [1, 30].

### Methodological aspects

Putting aside the theoretical aspects described above, one should also consider some of the methodological aspects of research efforts aimed at exploring the centrality hypothesis, which we will now discuss. First, we will describe extant studies examining the centrality hypothesis more indirectly, not focusing specifically on symptom change over time. We will then elaborate on a more direct approach used to examine the centrality hypothesis, describe findings of studies that have used it, and address some of the inherent limitations characterizing it.

While no study has yet to investigate the centrality hypothesis straightforwardly by examining the clinical efficacy of an intervention targeting pre-treatment central symptoms compared with an intervention targeting pre-treatment non-central symptoms, different studies have tried to elucidate the validity of the centrality hypothesis, or some of its assumptions, using different methodological approaches. Some have compared different features of networks constructed for the same sample at two different time-points, as symptoms were expected to differ between them. However, opposite and contrasting network connectivity-to-overall-symptoms associations emerged [53, 54]. Others have compared the baseline network structures (i.e., assessed at a single time-point) of two sub-samples of a single cohort “created” based on a difference in symptoms found at a later time-point (i.e., poor vs good treatment responders). Here, too, opposite result patterns were reached [55, 56]. Some have tried to address the centrality hypothesis by using simulation-aided procedures, showing that the removal of central nodes from a given network has no larger effect on the resultant network structure compared to removing nodes at random [57]. However, simulation studies can provide only indirect evidence with regard to the centrality hypothesis. Finally, others have examined whether centrality measures could *predict* clinical outcomes at a later time-point [33, 58]. While showing some positive findings, these studies did not examine *symptom change* over time, providing only indirect evidence for the centrality hypothesis. Furthermore, the latter study also found that the centrality-outcome relationships were not significantly stronger compared to the simple feature of symptom count [58].

While the aforementioned research has considerably advanced our knowledge in the field, only three studies to date were designed to more directly assess the centrality hypothesis as it relates to symptom change over time [18, 32, 34], with two examining the validity of pre-treatment central nodes in predicting symptom change over the course of treatment [18, 32]. All three studies used the same procedure developed by Robinaugh et al. [18]. The Robinaugh et al. procedure is based on the assumption that if nodes are causally connected, then changes in one node’s individual severity from one time-point to another (Δnode) would impact the severity of all remaining nodes of the network to which it is connected (summed up as Δnetwork [18]). Hence, a relation between Δnode and Δnetwork is assumed. Given that centrality identifies nodes with higher causal importance within a network, then changes in central nodes from one time-point to the next should cause proportionally greater changes in the rest of the network, compared to changes in less central nodes. Consequentially, centrality should be associated with the relation between Δnode and Δnetwork [18].

Examining the results of studies using the Robinaugh et al. procedure reveals mixed findings and some limitations characterizing each of them [18]. First, Robinaugh et al., examining complicated grief using a 13-item questionnaire among 195 participants, reported that all assessed centrality measures (e.g., strength, closeness, betweenness, expected influence) strongly correlated with the Δnode-Δnetwork association [18]. However, obtained results had large confidence intervals (e.g., for strength, *r*(11) = .66 [.18, .89]) lowering their specificity. Also, and most relevant to the present investigation, the authors did not investigate a treatment sample with pre- and post-treatment assessment, but rather a cohort from a longitudinal study of bereavement. Second, Rodebaugh et al., examining social anxiety using a 22-item measure in a sample of 244 patients undergoing treatment, also found a significant correlation between several centrality measures (strength, betweenness, and a composite centrality index) and the Δnode-Δnetwork association [32]. However, the observed effects failed to generalize to three additional social anxiety measures,^4^ a generalization that is to be expected under the centrality hypothesis. Moreover, infrequency of symptom endorsement (i.e., number of times the symptom was rated zero by participants), specifically chosen because it has no obvious causal effect on the Δnode-Δnetwork association, was not only found to be predictive, but also generalized across the other measures. Finally, Papini et al. examining posttraumatic symptoms using a 17-item questionnaire in a sample of 306 female patients with co-occurring substance use disorders and full or subthreshold PTSD, found two pre-treatment centrality measures (i.e., node strength and predictability) and one non-centrality node property (i.e., symptom severity) to be significantly correlated with the Δnode-Δnetwork association [34]. However, these measures were also found to be of limited robustness. Also, generalization to other measures or the effect of infrequency of symptom endorsement was not examined. Finally, a shared limitation of all three studies was the employment of a relatively small sample size which limits the stability of the network structure and the corresponding centrality metrics.^5^

While the Robinaugh et al. procedure is assumed to more directly examine the centrality hypothesis, some the procedure’s inherent limitations should be discussed, which may also explain the aforementioned mixed findings [18]. First, as pointed out by Rodebaugh et al., centrality measures are known to be affected by item properties like variance or ceiling effects [32, 59]. Thus, it may be that the predictiveness of centrality measures is simply driven by these simple, non-causal item properties. Second, (symptom) change is a second order concept that is inferred from differences obtained between constructs/networks at two (or more) different assessments, assumed to be the “same” [42, 60, 61]. However, the assumption of invariance has been mostly overlooked in the context of repeated cross-sectional network analyses. Third, as the measure used in this procedure is the correlation between node’s centrality and the Δnode-Δnetwork association, the number of nodes corresponds to the number of observations. Thus, the power of this analysis is a priori restricted by the number of nodes included in the examined network (assuming a constant effect size and alpha level). Consequentially, to reach an adequate statistical power of 0.8, a network must be constituted by at least 21 nodes for a strong-sized effect and 64 nodes for a medium-sized effect. However, psychopathology measures containing symptom checklists with 64 items rarely exist. Moreover, the precise estimation of centrality in a network containing 64 nodes will require a sample size of several hundred participants, limiting the contexts in which the hypothesis can be investigated using this approach. Consequently, investigations based on fewer nodes will not only have limited power but also result in imprecise values of the investigated correlation (i.e., large confidence intervals).

Taken together, prior experimental investigation of the validity of centrality measures as signaling symptom change has produced some mixed findings, with different methodologies, centrality measures, and effects used and examined across studies [33, 57, 58]. While the three studies using the Robinaugh et al. procedure, more directly examining the validity of central nodes in predicting treatment change, did show that centrality was partially successful in doing so, this was limited to the measure used to construct the network, not generalizing to other measures of the same examined psychopathology, which should be expected under the centrality hypothesis [18]. In addition, results also showed some simple non-centrality measures to outperform centrality measures.

### The empirical study

Notwithstanding the aforementioned methodological and theoretical arguments, if we do choose to assume that the centrality hypothesis is true, and the procedure by Robinaugh et al. [18] is, in principle, adequate for investigating the centrality hypothesis in the context of cross-sectional, between-subject context, then we should be able to reliably demonstrate (a) the predictive validity of centrality indices and (b) their generalizability to different measures of the same psychopathology (i.e., predictiveness across different questionnaires).

In this empirical part of our study, we aimed to test these two hypotheses in a large sample of PTSD patients (*N* = 710), assessed before and after treatment completion. To ensure comparability with previous work, we tested the centrality hypothesis using the same method applied by the three studies mentioned above. As different centrality measures were used in these studies, we chose to examine all those that were found to be predictive of the Δnode-Δnetwork association in any of the studies (i.e., strength, expected influence, and predictability). To test the generalizability of obtained results to other measures of the same psychopathology, explored only in one of the previous studies [32], we examined the predictability of included centrality indices using two measures of PTSD. We also repeated the above-described analyses examining two simple non-centrality symptom measures (i.e., mean symptom severity and infrequency of symptom endorsement), whose predictive properties are not based on causal assumptions deduced from network theory, to better tease apart predictiveness from causality. We chose measures that were used in previous studies but that have yielded mixed results [32, 34]. Finally, to examine the invariance assumption, we assessed the degree of invariance of both networks from before to after treatment by comparing the pre- and post-treatment networks.

In sum, here we examine the centrality hypothesis using (1) three centrality measures (i.e., strength, expected influence, predictability); (2) in a large sample of patients with the same primary disorder (*n* = 710); (3) assessed before and after treatment; (4) using two psychopathology measures of PTSD; (5) while also incorporating two simple non-causal node properties (i.e., mean symptom severity and infrequency of symptom endorsement).

## Methods

### Participants and procedure

Our empirical investigation is a secondary analysis of data collected prospectively between 2006 and 2014 at an outpatient clinic specialized in treating combat-related PTSD (Israel Defense Forces Unit for Treatment of Combat-Related PTSD). Participants were 710 treatment-seeking male veterans meeting diagnostic criteria for PTSD assessed via a semi-structured diagnostic interview based on the Diagnostic and Statistical Manual of Mental Disorders (DSM)-IV-TR [62], with all veterans being exposed to combat-related traumatic events (i.e., criterion A). A clinical diagnosis of PTSD was ascertained using the Clinician Administered PTSD Scale (CAPS-IV [63]), based on the F1/I2 item rule (i.e., Frequency > 1, Intensity> 2 [63]). Accordingly, PTSD diagnosis necessitated endorsing at least one re-experiencing symptom, three avoidance symptoms, and two hyperarousal symptoms. None of the veterans were receiving concurrent psychotherapy or pharmacotherapy elsewhere. Patients’ individual treatment plans were determined by the assessing clinician based on the clinical presentation of the patient (see Additional file 1: Table S1 for PTSD symptom severity per treatment type at pre- and post-treatment; for more details see [64]).

For the present study, all veterans for whom a complete assessment pre- and post-treatment was available (i.e., treatment completers) were included in the study. There were no veterans that completed their course of treatment without completing their post-treatment assessment. This resulted in a sample of 710 males out of the total 1795 (39.55%) veterans included in the original dataset. Consequentially, there was no missing data among the included participants. Participant characteristics are outlined in Table 1 (for a more detailed description of the original dataset, see [29]). The study was approved by the Ethics Committee of the IDF Medical Corps.

**Table 1:** Demographic characteristics of the sample (*n* = 710)

	*M*	*SD*
Age at assessment (years)	36.81	14.34
Age at event (years)	24.01	5.92
Education (years)	12.51	1.86
Family status (%)		
Single	45.8	–
Married	45.0	–
Divorced	8.9	–
Widowed	0.3	–
Number of children (*N*)	1.49	1.67
Employed (%)	60.3	–
Military occupation (%)		
Combat soldiers	84.8	–
Specialist military personnel	15.2	–
Officers (%)	8.8	–
Injured (%)	15.1	–
Past psychotherapy (%)	44.0	–
Past pharmacotherapy (%)	24.0	–
Treatment type (%)		
CBT	19.9	–
FGT	7.1	–
PDT	24.8	–
PGT	17.3	–
Pharmacotherapy	30.9	–
CAPS		
Pre-treatment	83.12	16.99
Post-treatment	59.48	26.21
PCL		
Pre-treatment	60.27	10.62
Post-treatment	48.28	15.32

*Note. PTSD* posttraumatic stress disorder, *CBT* cognitive behavior therapy, *TF-GT* trauma-focused group therapy, *PDT* psychodynamic therapy, *PGT* psychodynamic group therapy, *CAPS* Clinician-Administered PTSD Scale, *PCL* PTSD Checklist

### Measures

#### PTSD symptoms

PTSD symptoms at pre- and post-treatment were assessed using both a clinician-rated and a self-report measure. Clinician-rated PTSD symptoms, as defined by DSM-IV-TR [62], were assessed using the Clinician Administered PTSD Scale-IV and rated per the required guidelines [63]. Each of the 17 symptoms/items of the Clinician Administered PTSD Scale is rated separately on intensity and frequency on a 5-point Likert scale, ranging from 0 to 4, for an item total score of 0 to 8. Ratings are then summed yielding an estimate of three symptom clusters (i.e., Cluster B, Re-experiencing; Cluster C, Avoidance, and Numbing; and Cluster D, Hyperarousal), and an overall PTSD severity score. The Clinician Administered PTSD Scale is considered the gold standard for PTSD assessment, demonstrating excellent reliability, convergent and discriminant validity, diagnostic utility, and sensitivity to clinical change in military veterans and other populations, and has been extensively used in PTSD research [65, 66]. In the current sample, Cronbach’s *α* was 0.81 for pre-treatment assessment and 0.94 for the post-treatment assessment.

Self-reported PTSD symptoms were assessed using the PTSD Checklist for DSM-IV (PCL [67]). The PTSD Checklist is a 17-item self-report measure of PTSD symptom severity per DSM-IV. Each item assesses the extent to which the individual was bothered by the corresponding symptom during the last month using a 5-point scale ranging from 1 (“Not at all”) to 5 (“Extremely”), resulting in a total score ranging between 17 and 85. The PTSD Checklist has been shown to have good psychometric properties demonstrating high reliability and validity in veteran populations [68]. In the current sample, Cronbach’s *α* was 0.85 for pre-treatment assessment and 0.95 for the post-treatment assessment.

### Data analysis

The data analysis plan consisted of two efforts. We first conducted a replication of the analysis outlined by Papini et al. [34], and then performed an extension of their original analyses. All analyses were conducted in the *R* environment (Version 3.6.1 [69]). The resulting analytic *R* code is available in full in Additional Files (Additional file 3).

#### Replication analysis

The theoretical foundations of this analysis are outline above. The specific details of the actual analytic procedure conducted by Papini et al. are documented in detail also in their original publication [34]. As the original R code used was published by the authors, it enabled us to use the same code for the present replication attempt. Several minor changes to the code were introduced for the present analysis, none of which changed any of the steps required for the original analysis. These changes are documented as annotations in the R code, which is available in full as part of the Additional Files of this paper (Additional file 3).

Specifically, we estimated a network of pre-treatment symptoms using graphical lasso, calculated several centrality measures in this network (strength, expected influence, and predictability) and assessed network stability and reliability using the packages *qgraph* [70], *mgm* [71], and *bootnet* [72]. In the resulting network, nodes correspond to symptoms and edges represent partial correlations between them [72]. The Δnode-Δnetwork association was calculated as a correlation following the procedure developed by Robinaugh et al., which is outlined in detail above [18]. The predictiveness of centrality measures was then assessed by correlating the centrality metrics with the Δnode-Δnetwork association. Following Rodebaugh et al., we *z*-standardized all the included metrics and the Δnode-Δnetwork association prior to the correlation analysis [32]. A sensitivity analysis, correlating the non-standardized values, revealed similar results.

#### Extension analyses

We extended the analysis of Papini et al. in several important ways [34]. First, while Papini et al. used node’s strength and predictability, as well as symptom’s mean severity [34], at pre-treatment, we included two additional node metrics, namely, expected influence and infrequency of symptom endorsement, both of which were used in previous research examining the centrality hypothesis [18, 32]. Importantly, symptom severity and infrequency of symptom endorsement were explored as they both reflect node features that have no obvious causal properties (see above and [32] for more details), enabling to tease apart predictability and causality. Second, we aimed to assess if obtained results would also generalize to networks based on a different measure of the same psychopathology. Thus, we computed networks based on clinician-evaluated and self-reported symptom assessment (Clinician Administered PTSD Scale and PTSD Checklist, respectively) of the same patients at the same time-point. Third, we repeated these two analyses after removing the “amnesia” item (i.e., difficulties remembering different aspects of the traumatic experience), a known outlier in the phenomenology of PTSD [29, 30, 73, 74]. Finally, to examine the invariance assumption, we assessed the (in)variance of both networks across the two time-points (before and after treatment) by comparing the pre- and post-treatment networks using the *NetworkComparisonTest* [75] and by conducting community analyses in all four networks using the *walktrap algorithm* implemented in the *EGAnet* package [76].

In sum, the linear relations between five different node metrics (i.e., expected influence, strength, predictability, mean symptom severity, and infrequency of endorsement) and the Δnode-Δnetwork association were investigated using correlation analysis (Pearson coefficient), once in a network based on the Clinician Administered PTSD Scale and once in a network based on the PTSD Checklist. For each measure, this was conducted once with and once without the amnesia node. Thus, in total four analytic scenarios were conducted per node (i.e., Clinician Administered PTSD Scale and PTSD Checklist, each with and without amnesia). All *p* values were adjusted for multiple testing using the Benjamini-Hochberg procedure [77].

## Results

### PTSD networks and node metrics

The estimated networks based on the pre-treatment Clinician Administered PTSD Scale and PTSD Checklist data and the corresponding stability analyses are all shown in the Additional Files (Additional file 2: Figure S1 – S12). Briefly, the CS coefficients were above 0.7 in both networks (values above 0.5 indicate stable results). In both pre-treatment networks, *flashbacks* emerged as most influential while *amnesia* had the least expected influence. Overall, the results were comparable to network analyses of similar samples [29, 30]. The *NetworkComparisonTest* revealed that the structure of the Clinician Administered PTSD Scale and PTSD Checklist network both changed from pre- to post-treatment (both *p* < .001), which was supported also by the results of the community analyses (see Additional file 1: TableS2).

### Association of node metrics with symptom change

**Fig. 1:**
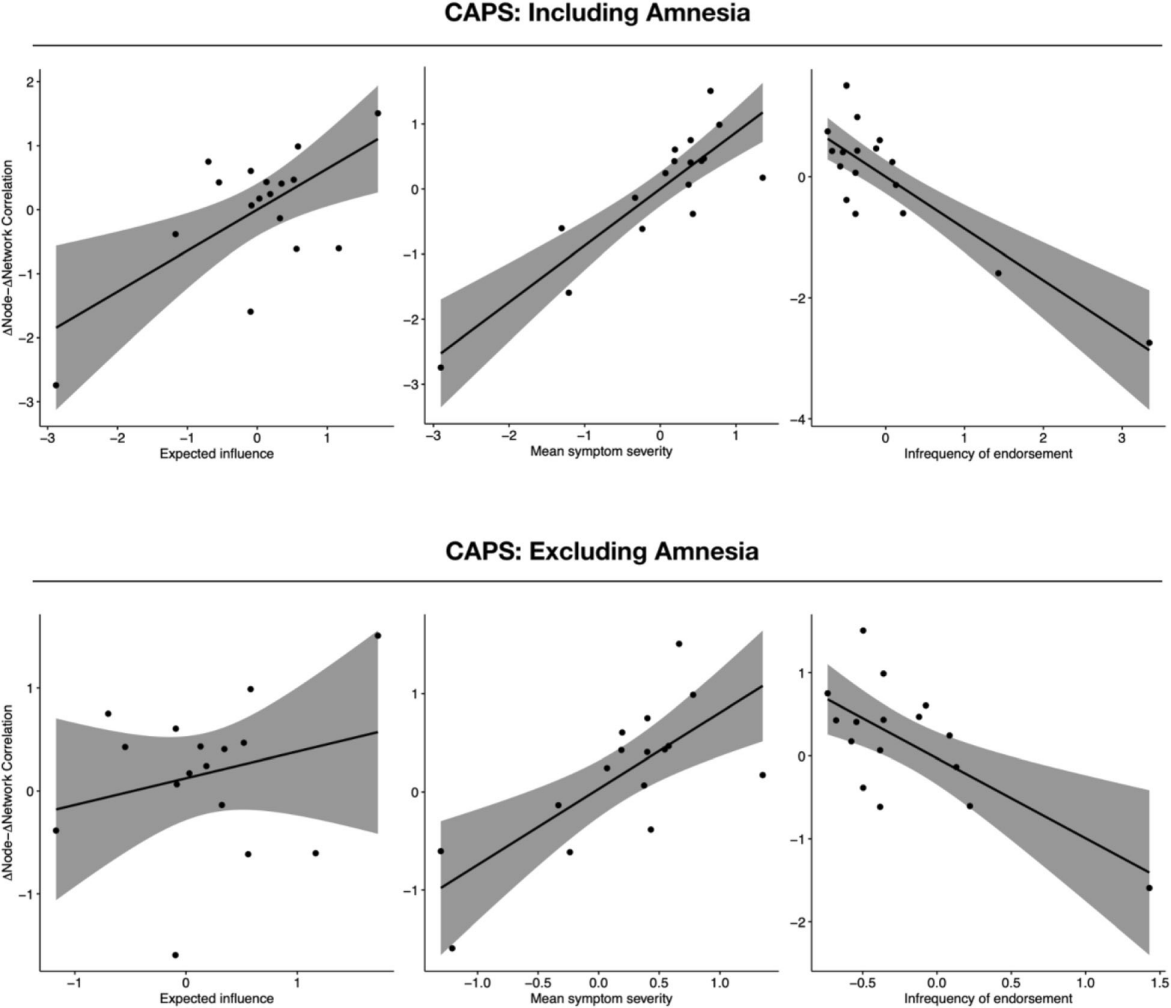
Scatterplots for the relation between the three significant node metrics and the Δnode-Δnetwork association for networks based on the Clinician-Administered PTSD Scale (CAPS) with and without the “amnesia” item

**Fig. 2:**
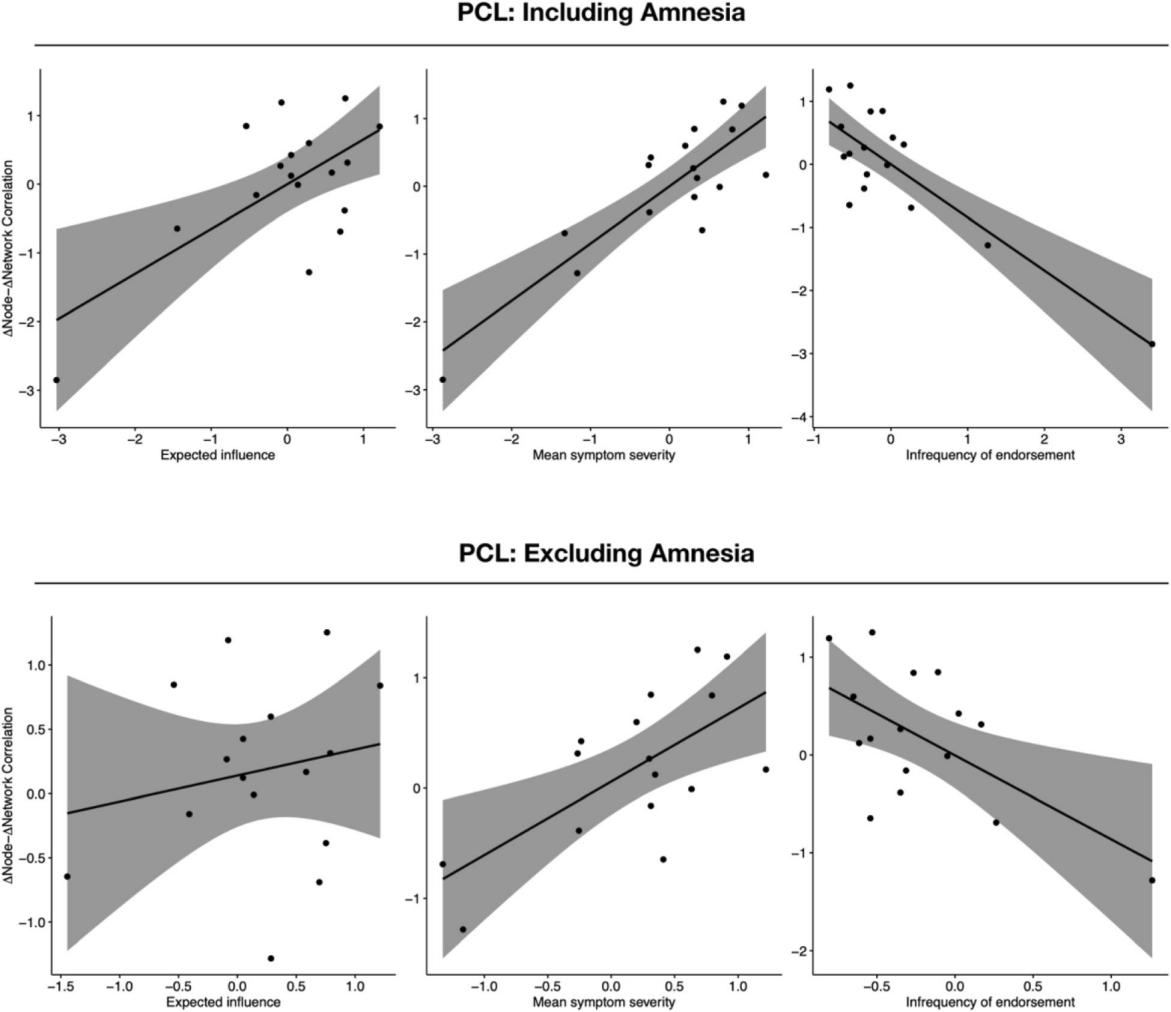
Scatterplots for the relation between the three significant node metrics and the Δnode-Δnetwork association for networks based on the PTSD Checklist (PCL) with and without the “amnesia” item

Table 2 outlines the correlation between all node metrics and the Δnode-Δnetwork association for both questionnaires (i.e., Clinician Administered PTSD Scale and PTSD Checklist) and for both datasets (with and without “amnesia”), including the correlations between item variance and the different centrality measure. Illustrative scatterplots of significant correlations for the Clinician Administered PTSD Scale and PTSD Checklist are shown in Figs. 1 and 2, respectively, and non-significant correlations in Additional file 2: Figure S13 and S14, respectively.

**Table 2:** The relationship between the Δnode-Δnetwork association and the assessed node metrics for networks based on the Clinician Administered PTSD Scale and the PTSD Checklist with or without the “amnesia” item. *p* values were adjusted for multiple testing

Questionnaire	Metric	All symptoms included			Amnesia excluded		
		*r (15)*	*95% CI*	*p*	*r (14)*	*95% CI*	*p*
CAPS	Strength^†^	.239	[−.273, .646]	.513	−.085	[−.557, .429]	.885
	EI	.641	[.231, .857]	.013	.246	[−.285, .661]	.513
	Predictability^†^	.435	[−.057, .758]	.135	−.051	[−.553, .456]	.885
	Mean severity	.870	[.669, .952]	.004	.727	[.362, .889]	.004
	Infrequency^†^	−.857	[−.947, −.639]	.004	−.690	[−.884, −.296]	.010
PCL	Strength^†^	.038	[−.451, .509]	.885	−.097	[−.566, .418]	.885
	EI	.653	[.251, .863]	.011	.186	[−.341, .624]	.805
	Predictability^†^	.449	[−.041, .764]	.129	−.040	[−.525, .465]	.885
	Mean severity	.844	[.612, .943]	.004	.659	[.250, .870]	.012
	Infrequency^†1^	−.843	[−.941, −.610]	.004	−.609	[−.848, −.162]	.024

*Note. r* Pearson correlation coefficient, *CAPS* Clinician-Administered PTSD Scale, *PCL* PTSD Checklist, *EI* expected influence; Infrequency = infrequency of endorsement, † = significant correlation between item variance and centrality measure, 1 = only in the network without “Amnesia”

When all symptoms were included, *mean symptom severity* and *infrequency of endorsement* were significantly correlated with the Δnode-Δnetwork association using both the Clinician Administered PTSD Scale and the PTSD Checklist, as was *expected influence*, albeit to a lesser extent. No significant findings emerged for *strength* and *predictability*. After excluding amnesia, only *mean symptom severity* and *infrequency of endorsement* remained significantly correlated with the Δnode-Δnetwork association using both PTSD measures. The point estimate of size of significant correlations varied between *r* = .878 for *mean symptom severity* in the Clinician Administered PTSD Scale network (including all items) and *r* = .659 for *mean symptom severity* in the PTSD Checklist network (excluding “amnesia”). Of note, the 95% confidence intervals of some of these significant effects were large (e.g., .250 to .870 for *mean symptom severity* in the PTSD Checklist network excluding amnesia; −.884 to −.296 for *infrequency of symptom endorsement* in the Clinician Administered PTSD Scale network excluding amnesia).

## Discussion

This article investigated the centrality hypothesis, namely, that centrality measures can be used to help identify causal influential nodes which may constitute valid targets for therapeutic interventions in cross-sectional, group-level networks. First, we presented several theoretical arguments that question the validity of the centrality hypothesis in psychopathology, suggesting this hypothesis to be built upon some unsubstantiated assumptions. Next, we addressed some methodological aspects of extant studies that used centrality measures as predictors of treatment change, while delineating several of their limitations. Finally, using pre- and post-treatment data collected from a large sample of 710 treatment-seeking patients with combat-related PTSD as an example, we conducted an empirical-guided analysis of the centrality hypothesis by replicating and extending previous research. Results pertaining to centrality measures and to simple non-causal node properties (i.e., non-centrality measures) guide our interpretation of the results.

Regarding our empirical analysis, our results only partially replicated previous findings, as only the expected influence centrality measure, but not node strength or predictability, was found to be significantly correlated with the Δnode-Δnetwork association. While the expected influence finding is in line with the results of Robinaugh et al., lack of findings for strength and predictability are at odds with previous studies [32, 34]. Our results are also in contrast with Rodebaugh et al. that found no generalization of the examined centrality measure to networks based on other questionnaires, while here the predictive effect of expected influence did generalize to the PTSD Checklist network. While these findings seem to provide some support for the centrality hypothesis, at least when considering expected influence, the observed effects (i.e., its predictive validity and generalization) completely disappeared after excluding amnesia, a well-recognized and documented outlier in the phenomenology of PTSD. Thus, the expected influence centrality measure only predicted the Δnode-Δnetwork association in two out of the four analytic scenarios, both of which included this known phenomenological outlier [30].

As for the additional non-centrality node features, results showed that both infrequency of symptom endorsement and symptom’s mean severity at pre-treatment reliably predicted the Δnode-Δnetwork association across measures (i.e., generalizability). Importantly, while expected influence also showed predictive validity and generalization, both of these non-centrality node features showed higher association strength with the Δnode-Δnetwork association across both measures. Moreover, as opposed to the expected influence centrality measure, after excluding amnesia this association remained significant as well as generalized. These findings are in line with the study of Rodebaugh et al., who also reported infrequency of symptom endorsement to reliably predict the Δnode-Δnetwork association across different questionnaires in a sample of patients with social anxiety disorder [32]. In contrast, findings are at odds with Papini et al. who reported no correlation between mean symptom severity and the Δnode-Δnetwork association examining PTSD symptoms [34]. However, Papini et al’s. study used a small sample with very different characteristics from the present sample, which might explain the divergence of results [34]. Specifically, their sample was comprised only of women with full or subthreshold PTSD as a comorbid condition with substance use disorder, from a multisite clinical trial, while here we used a large sample of clinically diagnosed men patients with PTSD following military-related traumatic events from a single site.

Taken together, results suggest that two simple item properties with no obvious causal influence significantly predicted the Δnode-Δnetwork association in all four analytic scenarios (network based on Clinician Administered PTSD Scale or PTSD Checklist, including or excluding “amnesia”), outperforming the traditional centrality measures. The larger sample size employed in the present study, compared with previous ones, strengthens the validity of current results. We would like emphasize at this juncture that the overarching goal of the present research was not to identify which item properties have best predictive capacities, but to use the predictiveness of centrality and non-centrality measures of the Δnode-Δnetwork association to examine the causal validity of the centrality hypothesis. Still, if one is solemnly interested in predicting the Δnode-Δnetwork association, it seems that current result would suggest using simple item properties. Interestingly, this latter suggestion adheres to the principle of Ockham’s razor, which can be paraphrased in this context as stating that if two models have equal predictive power, then the one with fewer assumptions (i.e., the less complex) should be preferred [78].

In sum, the three types of evidence presented above do not seem to lend strong support for using centrality measures in their current form to reveal treatment targets in cross-sectional, between-subject, psychopathological networks. Several important implications of current findings arise. First, echoing the assertions of Bringmann at al., while also acknowledging mental disorders as complex systems, it seems that centrality metrics currently used in psychopathology research, and their interpretation thereof, are based upon assumptions not met in the context of psychopathological research, raising the possibility of abandoning current measures or developing psychopathological research-specific ones [35]. Second, while most research under the network approach to psychopathology has been situated in the context of cross-sectional, between-subject, networks [1, 30], an increasing number of studies are now using novel idiographic, longitudinal designs trying to better elucidate the network structures of different psychopathologies. Hence, examining whether the centrality hypothesis holds under these new contexts is imperative. When doing so, we would strongly argue not just for testing the assumption empirically, but also for considering and elucidating the theoretical assumptions underlying the procedures chosen to test it. In any case, the specific context of the network investigated (e.g., directed or undirected, representing individual or group-level effects) will result in specific assumptions that will dictate not only the potential validity, but also the interpretation, of any applied centrality measure. Third, structural invariance of networks has been mainly thematized as an important assumption in the context of longitudinal networks based on frequent sampling of participants (e.g., several times daily [79]). Nevertheless, we think that the implications of model invariance (and violations thereof) for network analysis is imperative and as such should be more actively addressed in future research (e.g., as in [80]). Moreover, we believe that the “network research community” can benefit extremely from accumulated evidence and rigorous discussions held on this issue in related realms of research (e.g., [81]). Indeed, in the present study, the conducted *NetworkComparisonTest* and the community analyses implied that the structure of the present networks were not invariant from pre- to post-treatment. This further questions the validity of the procedure developed by Robinaugh et al., specifically the use of change scores in this context [18]. Finally, while the notion that influential nodes can be identified using empirical centrality measures is very appealing, the transition from a theoretical concept to a clinical application is not simple or straightforward. Theoretically-wise, like many other theories in psychopathological research [82], it seems that some aspects of the “network approach” are still mostly narrative, including several underlying, unspecified assumptions, which are then endowed to derived hypotheses. Indeed, a recent work indicates that the current data-driven network models fail to infer structures needed for the development of formal theories of psychopathologies [83]. Considering the centrality hypothesis specifically, and as outlined above, it still remains mostly unclear under which specific circumstances (e.g., study design, centrality measure) the centrality hypothesis, as it is currently formulated, should hold, making it an ill-defined hypothesis. This, in turn, hinders the accumulation of supporting or negating evidence, which is a necessary step before advancing to explore its clinical application. Hence, we believe that present findings highlight the need for a more formal definition of the “network approach”, and its derived hypotheses, including the centrality hypothesis. A potential road map for bridging the gap between theoretical and empirical models under a complex systems (and network) approach to psychopathology has been recently outlined [83], which might help network-based research move forward.

Our article is not without limitations. First, as stated above, we empirically tested the centrality hypothesis under a specific context (cross-sectional, between-subject networks), using a specific analytic approach, and a specific sample (PTSD patients). The fact that the present analysis was based on between-subject data (in accordance with the three previous studies [18, 32, 34]) is a major limitation in terms of testing the centrality hypothesis as a whole. However, this is also a strength of the present manuscript. Given that between-subject networks are still the most prevalent kind of psychopathological networks, the present study clearly suggests that both authors and readers of such studies be especially cautious when referring to the centrality hypothesis in interpreting obtained results. Also, while the Papini et al. study also examined PTSD, the sample used was very different compared with the present one [34]. Hence, future research in different contexts using different analytic approaches in different disorders/datasets should be conducted to substantiate current findings. Nevertheless, we believe that the presented complementary theoretical arguments hold independently of the specific context or the analytic approach taken. Second, and related to the previous point, the sample used in our empirical-guided analysis consisted entirely of treatment-seeking patients, all with a clinical diagnosis of PTSD based on DSM-IV. Hence, Berkson’s bias may apply, potentially introducing spurious associations between symptoms and therefore biasing centrality as well [84]. However, this bias would come into play in all network studies using clinical samples characterized by a high mean symptom severity (hence clinical). Thus, the potential effects of Berkson’s bias in clinical samples only serves to strengthen our previously outlined argument that methodical choices impact network structure, confounding the potential of centrality measures to reveal true causal influential nodes. The fact that for the field of psychopathology clinical samples are the population of interest makes the Berkson’s bias almost inevitable, completely undermining the utility of cross-sectional data in identifying treatment targets using the network approach to psychopathology. Third, although we presented complimentary theoretical arguments restricting the validity of the centrality hypothesis, we did not present additional logical or mathematical arguments to prove that the hypothesis is *in principle* false, a claim we do not make. Fourth, our results might have been affected by the different treatment regimens participants were assigned to, as different treatments likely affect individual symptom in a unique way. Nevertheless, as the centrality hypothesis is supposed to be valid independently of the chosen treatment, we do not consider this a major limitation. Still, future research could replicate the present one using a more homogeneous treatment modality group.

Notwithstanding these limitations, the present study also has several strengths. First, methodological, theoretical, and empirical efforts were introduced. Second, the empirical analysis was based on the largest sample size compared with previous studies, while also addressing some of their limitations (e.g., incorporating several centrality measures, including non-centrality simple features, examining generalization and invariance). Still, future research could investigate if and how the outlined arguments also apply to other empirical contexts beside cross-sectional and PTSD, as well as to other network-based metrics, for example bridge centrality or network density.

## Conclusion

In conclusion, the current study did not lend support for using centrality measures in their current form to reveal treatment targets in cross-sectional, between-subject networks. Moreover, simple non-causal item properties outperformed centrality measures. Several theoretical arguments also challenge the validity of the centrality hypothesis in this context. Thus, the centrality hypothesis in its current form seems to be too ill-defined to grasp the complexity of mental disorders in cross-sectional, between-subject networks, unless reformulated on a more solid theoretical and empirical ground.

## Supplementary information

**Additional file 1: Table S1.** PTSD symptom severity per treatment type at pre and post treatment. **Table S2.** Communities detected using the walktrap algorithm.

**Additional file 2: Figure S1.** The PTSD symptom network based on CAPS assessment at pre-treatment. **Figure S2.** The PTSD symptom network based on CAPS assessment at post-treatment. **Figure S3.** The PTSD symptom network based on PCL assessment at pre-treatment. **Figure S4.** The PTSD symptom network based on PCL assessment at post-treatment. **Figure S5.** Bootstrap node strength difference test of the nodes of the CAPS pre-treatment network. **Figure S6.** Bootstrap node strength difference test of the nodes of the PCL pre-treatment network (Figure S2). **Figure S7.** Bootstrap edge weights difference test of the nodes of the pre-treatment CAPS network (Figure S1). **Figure SS8.** Bootstrap edge weights difference test of the nodes of the pre-treatment PCL network (Figure S2). **Figure S9.** Bootstrap 95% confidence intervals for estimated edge weights for the pre-treatment CAPS network (Figure S1). Figure S10. Bootstrap 95% confidence intervals for estimated edge weights for the pre-treatment PCL network (Figure S2). **Figure S11.** Strength and Expected Influence of the pre-treatment CAPS network (Figure S1). **Figure S12.** Strength and Expected Influence of the pre-treatment PCL network (Figure S3). **Figure S13.** Scatterplots for the relationship between the two non-significant node metrics and the Δnode - Δnetwork association for networks based on the CAPS with and without the “amnesia” item. **Figure S14.** Scatterplots for the relationship between the two non-significant node metrics and the Δnode - Δnetwork association for networks based on the PTSD Checklist (PCL) with and without the “amnesia” item.

**Additional file 3. ***R* code used in the presented analyses.

## Data Availability

The datasets generated and/or analyzed during the current study are not publicly available due to Israel Defense Forces’ (IDF’s) Information Security restrictions but are available from the corresponding author on reasonable request and with permission of the IDF. The R code used to analyze the data for the current study is available in full as part of the supplementary materials of this paper.

## Abbreviations

DSM: Diagnostic and Statistical Manual of Mental Disorders
PTSD: Posttraumatic stress disorder

## Footnotes

1 Still, some authors are much more cautious when interpreting centrality indexes and when emphasizing their clinical meaning (e.g., [28][29]).

2 While this gap between the theoretically proposed mechanisms (within individuals) and the most often used study designs for their investigation (group-level) in psychopathological research is not unique to network analysis [40], it has special relevance for the network approach to psychopathology as within-individual processes are at the core of this approach. Indeed, it has been suggested that one of the main aims of cross-sectional group-level-based network studies is to generate hypotheses about individual effects, which might be recovered from group-level networks [6]. Hence, network analysis is especially affected by the choice of the level of investigation.

3 While all research efforts employing a data processing pipeline, as used for example in imaging-based research, might be similarly affected by the specific decisions made in setting up the pipeline, in other fields standardized protocols for data processing exist (e.g., genome wide association studies; [51]). However, such processing standards are mostly lacking in network analysis

4 A composite measure of centrality, convoluting two centrality metrics obtained in the pretreatment network based on sample A and questionnaire X predicted the Δnode - Δnetwork association in sample B based on questionnaire X. However, the centrality measure did not predict the Δnode - Δnetwork association in sample B based on questionnaire Y and Z.

5 As outlined by Robinaugh et al., it is important to mention that a strong test of the centrality hypothesis would have been to examine change in networks within single people [18]. However, there has been no real rigorous test of this hypothesis, with actual research using this procedure in idiographic study designs yet.
