# Supplementary material for: On the validity of the centrality hypothesis in cross-sectional between-subject networks of psychopathology
Source: BMC Med. 2020 Oct 12;18:297. doi: 10.1186/s12916-020-01740-5 (PMC7549218; doi:10.1186/s12916-020-01740-5)
Supplement: Supplementary file 1 — Additional file 1: Table S1. PTSD symptom severity per treatment type at pre and post treatment. Table S2. Communities detected using the walktrap algorithm. [file 12916_2020_1740_MOESM1_ESM.docx]

**Additional File 1: Tables**

**Table S1.**

*PTSD symptom severity per treatment type at pre- and post-treatment*

| Treatment type | Pre-treatment | | Post-treatment | | *P* value |
| --- | --- | --- | --- | --- | --- |
| Measure | *M* | *SD* | *M* | *SD* |  |
| CBT |  |  |  |  |  |
| CAPS | 78.48 | 18.27 | 58.31 | 25.64 | <.0001 |
| PCL | 59.04 | 10.21 | 48.70 | 14.87 | <.0001 |
| TF-GT |  |  |  |  |  |
| CAPS | 82.22 | 10.59 | 51.36 | 23.76 | <.0001 |
| PCL | 49.34 | 12.37 | 34.42 | 11.64 | <.0001 |
| PDT |  |  |  |  |  |
| CAPS | 78.71 | 17.72 | 51.83 | 25.81 | <.0001 |
| PCL | 58.23 | 10.96 | 44.54 | 14.41 | <.0001 |
| PGT |  |  |  |  |  |
| CAPS | 84.77 | 18.94 | 70.67 | 25.29 | <.0001 |
| PCL | 61.33 | 10.84 | 54.75 | 13.92 | <.0001 |
| Pharmacotherapy |  |  |  |  |  |
| CAPS | 88.75 | 13.23 | 62.20 | 25.25 | <.0001 |
| PCL | 64.23 | 8.29 | 50.68 | 15.06 | <.0001 |

*Note.* PTSD = Posttraumatic Stress Disorder; CBT = Cognitive Behavior Therapy; TF-GT = Trauma-Focused Group Therapy; PDT = Psychodynamic Therapy; PGT = Psychodynamic Group Therapy; CAPS = Clinician Administered PTSD Scale; PCL = PTSD Checklist

Table S2.

*Communities detected using the walktrap algorithm*

|  | CAPS | |  | PCL | |  |  |
| --- | --- | --- | --- | --- | --- | --- | --- |
| Variable | Pre-treatment | Post-treatment |  | Pre-treatment | Post-treatment |  |  |
| INT | 1 | 1 |  | 2 | 2 |  |  |
| FLA | 1 | 1 |  | 2 | 2 |  |  |
| PHY | 1 | 1 |  | 2 | 2 |  |  |
| AMN | 1 | 2 |  | 2 | 1 |  |  |
| DTA | 1 | 2 |  | 2 | 2 |  |  |
| STR | 1 | 3 |  | 2 | 1 |  |  |
| DRE | 2 | 1 |  | 1 | 2 |  |  |
| UPS | 2 | 1 |  | 1 | 2 |  |  |
| AVT | 2 | 1 |  | 1 | 2 |  |  |
| AVS | 2 | 1 |  | 1 | 2 |  |  |
| SLE | 2 | 1 |  | 1 | 2 |  |  |
| HYP | 2 | 3 |  | 1 | 1 |  |  |
| DIS | 3 | 2 |  | 1 | 1 |  |  |
| NUM | 3 | 2 |  | 1 | 1 |  |  |
| FUT | 3 | 2 |  | 1 | 1 |  |  |
| ANG | 3 | 1 |  | 2 | 1 |  |  |
| CON | 3 | 3 |  | 2 | 1 |  |  |
| Number of communities | 3 | 3 |  | 2 | 2 |  |  |
|  |  |  |  |  |  |  |  |

*Note.* The numbers indicate the community, to which an item belongs
