# Supplementary material for: On the validity of the centrality hypothesis in cross-sectional between-subject networks of psychopathology
Source: BMC Med. 2020 Oct 12;18:297. doi: 10.1186/s12916-020-01740-5 (PMC7549218; doi:10.1186/s12916-020-01740-5)
Supplement: Supplementary file 2 — Additional file 2: Figure S1. The PTSD symptom network based on CAPS assessment at pre-treatment. Figure S2. The PTSD symptom network based on CAPS assessment at post-treatment. Figure S3. The PTSD symptom network based on PCL assessment at pre-treatment. Figure S4. The PTSD symptom network based on PCL assessment at post-treatment. Figure S5. Bootstrap node strength difference test of the nodes of the CAPS pre-treatment network. Figure S6. Bootstrap node strength difference test of the nodes of the PCL pre-treatment network (Figure S2). Figure S7. Bootstrap edge weights difference test of the nodes of the pre-treatment CAPS network (Figure S1). Figure SS8. Bootstrap edge weights difference test of the nodes of the pre-treatment PCL network (Figure S2). Figure S9. Bootstrap 95% confidence intervals for estimated edge weights for the pre-treatment CAPS network (Figure S1). Figure S10. Bootstrap 95% confidence intervals for estimated edge weights for the pre-treatment PCL network (Figure S2). Figure S11. Strength and Expected Influence of the pre-treatment CAPS network (Figure S1). Figure S12. Strength and Expected Influence of the pre-treatment PCL network (Figure S3). Figure S13. Scatterplots for the relationship between the two non-significant node metrics and the Δnode - Δnetwork association for networks based on the CAPS with and without the “amnesia” item. Figure S14. Scatterplots for the relationship between the two non-significant node metrics and the Δnode - Δnetwork association for networks based on the PTSD Checklist (PCL) with and without the “amnesia” item. [file 12916_2020_1740_MOESM2_ESM.docx]

**Additional File 2: Figures**

Figure S1.

*The PTSD symptom network based on CAPS assessment at pre-treatment.*

*Note.* The network visualization represents: node size = mean symptom severity in the sample; edge thickness = strengths of the regularized partial correlation (blue = positive, red = negative correlations); shaded area of the node perimeter = predictability; node color = DSM-IV clusters of PTSD (re-experiencing = green, avoidance = orange, hyperarousal = blue).

Figure S2.

*The PTSD symptom network based on CAPS assessment at post-treatment.*

*Note.* The network visualization represents: node size = mean symptom severity in the sample; edge thickness = strengths of the regularized partial correlation (blue = positive, red = negative correlations); shaded area of the node perimeter = predictability; node color = DSM-IV clusters of PTSD (re-experiencing = green, avoidance = orange, hyperarousal = blue).

Figure S3.

*The PTSD symptom network based on PCL assessment at pre-treatment.*

*Note.* The network visualization represents: node size = mean symptom severity in the sample; edge thickness = strengths of the regularized partial correlation (blue = positive, red = negative correlations); shaded area of the node perimeter = predictability; node color = DSM-IV clusters of PTSD (re-experiencing = green, avoidance = orange, hyperarousal = blue).

Figure S4.

*The PTSD symptom network based on PCL assessment at post-treatment.*

*Note.* The network visualization represents: node size = mean symptom severity in the sample; edge thickness = strengths of the regularized partial correlation (blue = positive, red = negative correlations); shaded area of the node perimeter = predictability; node color = DSM-IV clusters of PTSD (re-experiencing = green, avoidance = orange, hyperarousal = blue).

Figure S5.

*Bootstrap node strength difference test of the nodes of the CAPS pre-treatment network (Figure S1).*

*Note*. Black boxes indicate significant differences between two nodes’ strength. Non-significant differences. The value of node strength is indicated by the number in the white boxes.

Figure S6.

*Bootstrap node strength difference test of the nodes of the PCL pre-treatment network (Figure S2).*

*Note*. Black boxes indicate significant differences between two nodes’ strength. Non-significant differences. The value of node strength is indicated by the number in the white boxes.

Figure S7.

*Bootstrap edge weights difference test of the nodes of the pre-treatment CAPS network (Figure S1).*

*Note*. Black boxes indicate significant differences between two nodes’ strength. Non-significant differences.

Figure S8.

*Bootstrap edge weights difference test of the nodes of the pre-treatment PCL network (Figure S2).*

*Note*. Black boxes indicate significant differences between two nodes’ strength. Non-significant differences.

Figure S9.

*Bootstrap 95% confidence intervals for estimated edge weights for the pre-treatment CAPS network (Figure S1).*


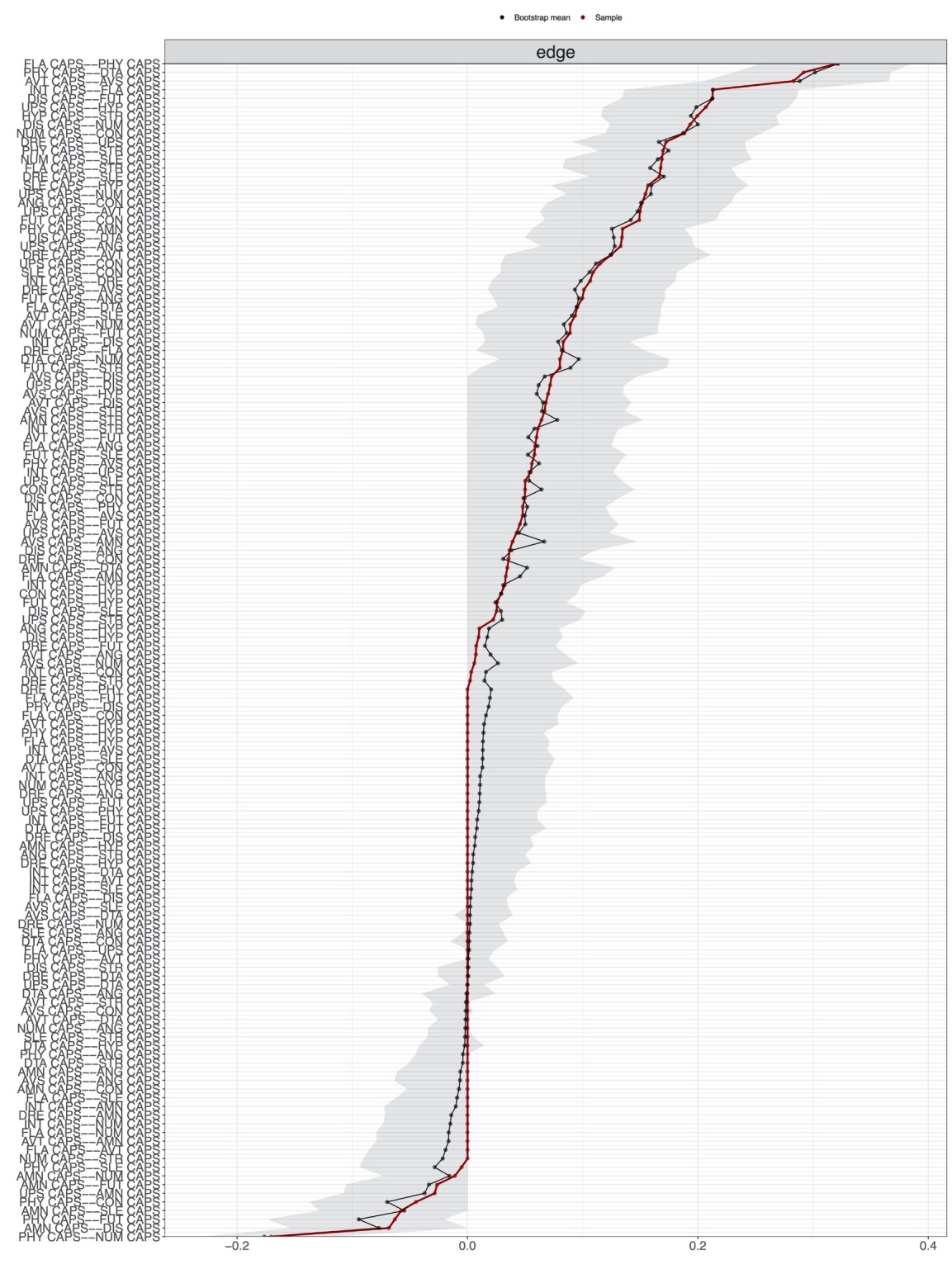


*Note*. Edges are represented by horizontal lines, edge weights by the red line. The 95% confidence intervals are indicated by the grey area. The black dots connected by the black line represent the bootstrapped means.

Figure S10.

*Bootstrap 95% confidence intervals for estimated edge weights for the pre-treatment PCL network (Figure S2).*


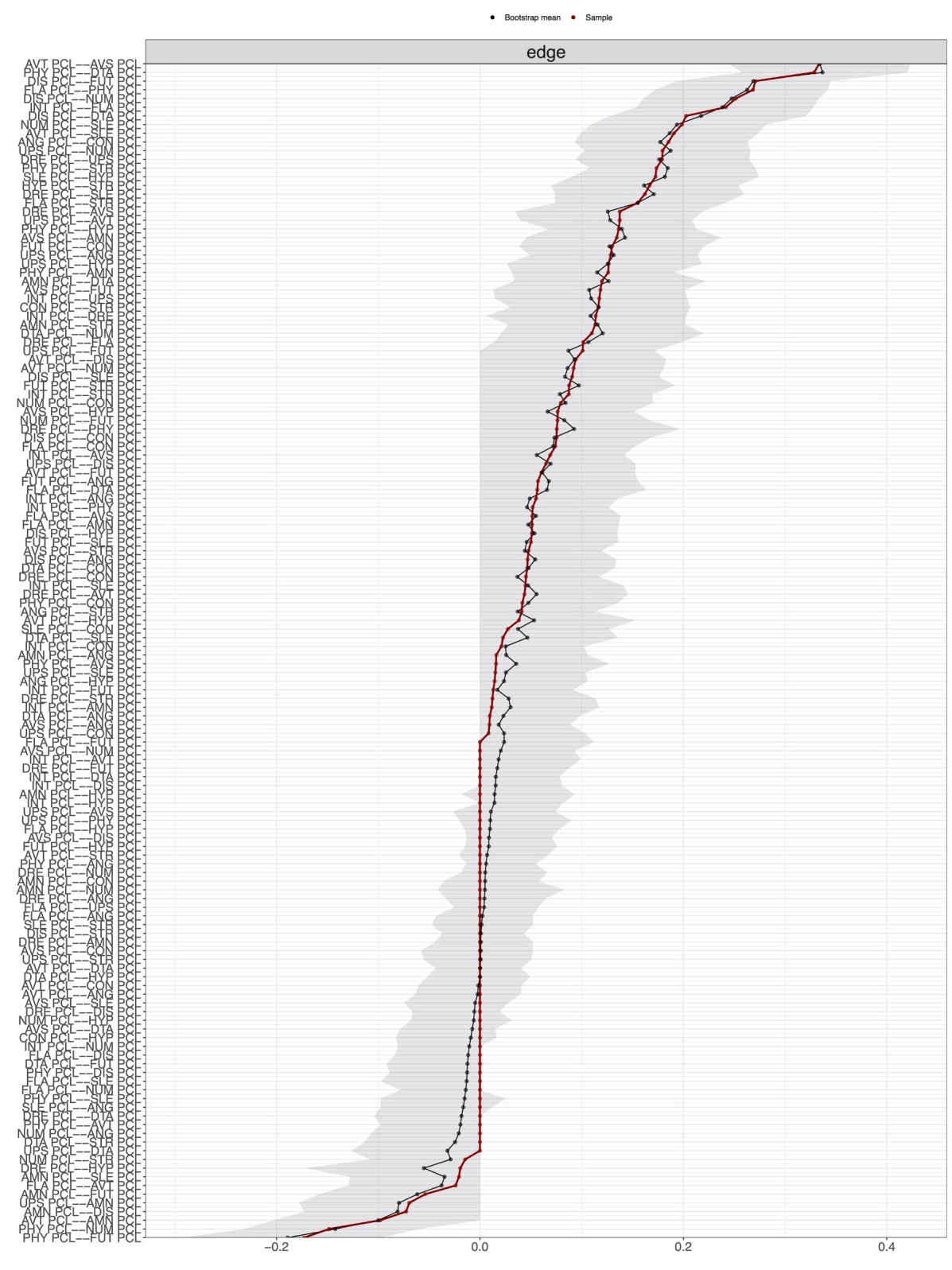


*Note*. Edges are represented by horizontal lines, edge weights by the red line. The 95% confidence intervals are indicated by the grey area. The black dots connected by the black line represent the bootstrapped means.

Figure S11.

*Strength and Expected Influence of the pre-treatment CAPS network (Figure S1).*

.

Figure S12.

*Strength and Expected Influence of the pre-treatment PCL network (Figure S2).*

Figure S13.
*Scatterplots for the relationship between the two non-significant node metrics and the Δnode - Δnetwork association for networks based on the CAPS with and without the “amnesia” item.*

*
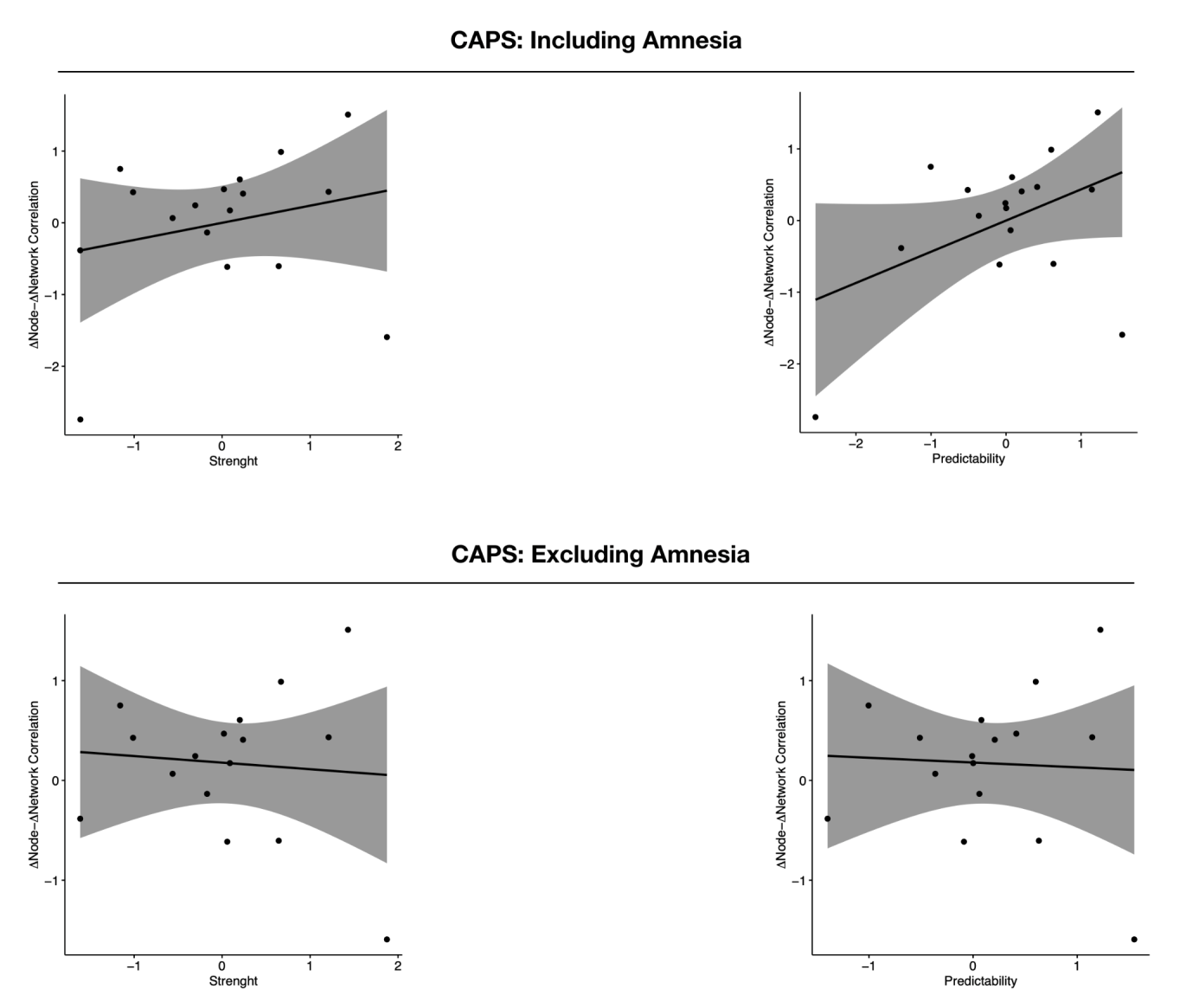
*

*Note.* The linear relationship is quantified using the Pearson correlation coefficient. The gray area corresponds to 95% confidence-intervals.

Figure S14.
*Scatterplots for the relationship between the two non-significant node metrics and the Δnode - Δnetwork association for networks based on the PTSD Checklist (PCL) with and without the “amnesia” item.
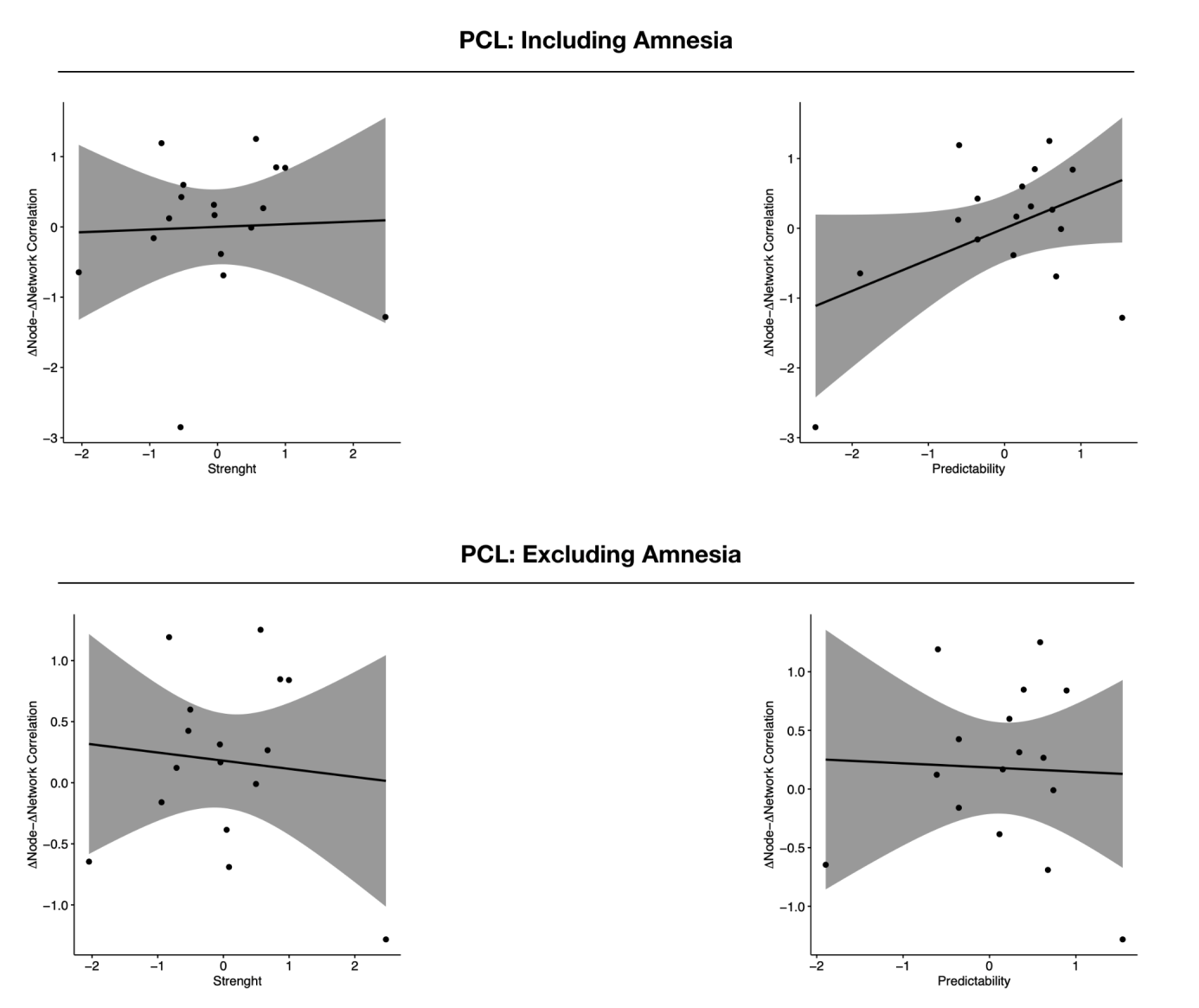
*

*Note.* The linear relationship is quantified using the Pearson correlation coefficient. The gray area corresponds to 95% confidence-intervals.
